# Gas6 Ameliorates Inflammatory Response and Apoptosis in Bleomycin-Induced Acute Lung Injury

**DOI:** 10.3390/biomedicines9111674

**Published:** 2021-11-12

**Authors:** Bo-Min Kim, Ye-Ji Lee, Youn-Hee Choi, Eun-Mi Park, Jihee Lee Kang

**Affiliations:** 1 Department of Physiology, College of Medicine, Ewha Womans University, Seoul 07804, Korea; qhals96@ewhain.net (B.-M.K.); shyzizibe@naver.com (Y.-J.L.); yc@ewha.ac.kr (Y.-H.C.); 2 Inflammation-Cancer Microenvironment Research Center, College of Medicine, Ewha Womans University, Seoul 07804, Korea; 3 Department of Pharmacology, College of Medicine, Ewha Womans University, Seoul 07804, Korea; empark@ewha.ac.kr

**Keywords:** Gas6, bleomycin, inflammation, apoptosis, efferocytosis

## Abstract

Acute lung injury (ALI) is characterized by alveolar damage, lung edema, and exacerbated inflammatory response. Growth arrest-specific protein 6 (Gas6) mediates many different functions, including cell survival, proliferation, inflammatory signaling, and apoptotic cell clearance (efferocytosis). The role of Gas6 in bleomycin (BLM)-induced ALI is unknown. We investigated whether exogenous administration of mouse recombinant Gas6 (rGas6) has anti-inflammatory and anti-apoptotic effects on BLM-induced ALI. Compared to mice treated with only BLM, the administration of rGas6 reduced the secretion of proinflammatory cytokines, including tumor necrosis factor-α, interleukin-1β, and macrophage inflammatory protein-2, and increased the secretion of hepatocyte growth factor in bronchoalveolar lavage (BAL) fluid. rGas6 administration also reduced BLM-induced inflammation and apoptosis as evidenced by reduced neutrophil recruitment into the lungs, total protein levels in BAL fluid, caspase-3 activity, and TUNEL-positive lung cells in lung tissue. Apoptotic cell clearance by alveolar macrophages was also enhanced in mice treated with both BLM and rGas6 compared with mice treated with only BLM. rGas6 also had pro-resolving and anti-apoptotic effects in mouse bone marrow-derived macrophages and alveolar epithelial cell lines stimulated with BLM in vitro. These findings indicate that rGas6 may play a protective role in BLM-induced ALI.

## 1. Introduction

Acute lung injury (ALI), a critical illness with high morbidity and mortality, is an intense pulmonary inflammatory response characterized by neutrophil recruitment, interstitial edema, disruption of epithelial integrity, and parenchymal injury [1]. The injurious event concomitantly activates a fibroproliferative response, which leads to increased fibroblast proliferation and extracellular matrix synthesis [2]. Bleomycin (BLM) is a chemotherapeutic agent used to treat different types of cancer, but one of its side effects is pulmonary toxicity [3,4,5]. BLM causes an interstitial pneumonitis that can lead to fibrosis [6]. Because the development of fibrosis is directly influenced by the extent of the initial lung injury, it is critical to understand the early changes caused by BLM [7]. Despite improvements in supportive care, the overall mortality rate of ALI patients is still nearly 40% [2,8,9]. Therefore, there is a critical need to investigate novel strategies to resolve ALI. Rodent models of early-stage, BLM-induced lung injury share many histologic similarities with clinical ALI in humans [10]. Thus, rodent models are important tools for investigating early changes induced by BLM.

Growth arrest-specific protein 6 (Gas6) belongs structurally to the family of plasma vitamin K-dependent proteins. Gas6 binds to TAM (Tyro3, Axl, and Mer) receptors, showing the highest affinity for binding Axl, followed by Tyro3, and then Mer [11]. Ligand binding to the immunoglobulin-like domains of TAM receptors triggers receptor dimerization, the autophosphorylation of an intracellular tyrosine, and signal transduction through different pathways. These pathways include mitogen-activated protein kinase (MAPK), phosphoinositide 3-kinase–AKT (PI3K/AKT), Janus kinase/signal transducers and activators of transcription (JAK/STAT), extracellular signal-regulated kinase (ERK), and nuclear factor-kappaB (NF-κB) pathways [12,13,14,15]. TAM activation by Gas6 mediates many different functions, including cell survival, proliferation, inflammatory signaling, apoptotic cell clearance (efferocytosis), differentiation, adhesion, migration, platelet function, and thrombus stabilization [16,17,18,19,20,21,22,23,24,25]. Through these functions, TAM signaling plays an important role in modulating the innate immune response. Furthermore, we previously demonstrated that Gas6 directly and indirectly contributes to alveolar epithelial cell homeostasis by regulating proliferation, tissue repair, and the epithelial–mesenchymal transition (EMT) [26,27].

Previous research suggests that Gas6 signaling has a protective role in mice models of multi-organ dysfunction syndrome, ALI in sepsis, and ischemia/reperfusion-induced ALI [28,29,30,31]. In contrast, Gas6 or Mer deficiency is protective against silica-induced lung inflammation and fibrosis in mice [32]. Furthermore, a recent study also demonstrates that targeting Gas6 and TAM receptors with either specific antibodies directed at Gas6 or Axl or with small-molecule TAM inhibitors attenuates the activation of pulmonary fibroblasts in idiopathic pulmonary fibrosis [33]. On the other hand, exogenous human protein S (hPS), the other TAM ligand, inhibits BLM-induced lung fibrosis and apoptosis of human alveolar epithelial cells [34]. However, whether exogenous administration of Gas6 has a protective or harmful role in BLM-induced ALI is largely unknown.

In the present study, we focused on the ALI induced during the 7 days after a BLM challenge when there is an enhanced inflammatory response and alveolar epithelial damage, but fibrosis has not yet been established [35,36,37]. The effect of rGas6 treatment was evaluated by assessing mouse inflammatory response, alveolar injury, and the efferocytic ability of alveolar macrophages after the BLM challenge. We also evaluated the in vitro inhibitory effects of rGas6 on inflammatory cytokine production in mouse bone marrow-derived macrophages (BMDMs), as well as its anti-apoptotic effects on alveolar epithelial cell lines after stimulation with BLM.

## 2. Materials and Methods

### 2.1. Reagents

Mouse rGas6 (986-GS) was purchased from R&D Systems (Minneapolis, MN, USA). BLM, paraformaldehyde, and LPS (*Escherichia coli* LPS, 055:B5) were purchased from Sigma-Aldrich (St Louis, MO, USA). The antibodies used in this study were the following: anti-phospho-Akt (sc-7985-R), anti-Akt (sc-8312), and anti-β-actin from Santa Cruz Biotechnology (Santa Cruz, CA, USA), as well as cleaved caspase-3 antibody (#9661) from Cell Signaling Technology (Danvers, MA, USA).

### 2.2. Animal Protocols

Specific pathogen-free male C57Bl/6 mice (Orient Bio, Sungnam, Korea) weighing between 20 and 22 g were used in all experiments. The Animal Care Committee of the Ewha Medical Research Institute approved the experimental protocol (ESM 17-0369). We administered BLM (5 U/kg in 30 μL) by mouse pharyngeal aspiration [37,38]. rGas6 (50 μg/kg) or saline treatment (BLM + Sal) was given intraperitoneally 1 h before BLM treatment, and then once a day thereafter [30,31]. Mice were euthanized on day 1, 2, or 7 following BLM treatment.

### 2.3. BAL Fluid Analysis and Differential Cell Counts

BAL was performed through a tracheal cannula using 0.7 mL aliquots of ice-cold Ca2+/Mg2+-free phosphate-buffered medium (145 mM NaCl, 5 mM KCl, 1.9 mM NaH2PO4, 9.35 mM Na2HPO4, and 5.5 mM dextrose, pH 7.4) for a total of 3.5 mL per mouse. BAL samples were centrifuged at 500× *g* for 5 min at 4 °C, and cell pellets were washed and resuspended in phosphate-buffered medium. The number of neutrophils and alveolar macrophages was determined according to their unique cell diameter, using an electronic Coulter counter fitted with a cell-sizing analyzer (Coulter Model ZBI with a channelizer 256; Coulter Electronics, Bedfordshire, UK) [39,40]. In addition, stained BAL cytospins with the Diff-Quik kit (Dade Behring, Newark, DE, USA) for differential of BAL alveolar macrophages and neutrophils [41] and assessment of phagocytic indices [36,42] were also performed. The phagocytic index was calculated using the following formula: [(number of apoptotic bodies)/(200 total macrophages)] × 100. After BAL, lungs were removed, immediately frozen in liquid nitrogen, and stored at −70 °C.

### 2.4. Enzyme-Linked Immunosorbent Assay (ELISA)

The concentrations of tumor necrosis factor-α (TNF-α), interleukin-1β (IL-1β), macrophage inflammatory protein-2 (MIP-2), and hepatocyte growth factor (HGF) in BAL fluid and cell culture samples were measured by ELISA kits as per the manufacturer’s instructions (R&D Systems, Minneapolis, MN, USA).

### 2.5. Total Protein Concentration

Protein concentrations of the BAL samples were used as indicators of blood–pulmonary epithelial cell barrier integrity. Total protein content in BAL fluid was measured according to the manufacturer’s protocols (BCA protein assay kit, Thermo Scientific, Rockford, IL, USA).

### 2.6. Quantitative Real-Time PCR (qPCR)

Total RNA was isolated from lung tissue using an Easy Spin RNA extraction kit (Intron, Seongnam Gyeonggi-do, South Korea) according to the manufacturer’s instructions. cDNA was generated using a ReverTraAce qPCR RT Master Mix (Toyobo, Osaka, Japan). Gene expression was analyzed using qPCR on a StepOnePlus system (Applied Biosystems, Life Technologies, Carlsbad, CA, USA). Primer sets for PCR-based amplifications were designed using Primer Express software. The primers used were as follows (name: forward primer, reverse primer): TNF-α: 5′-CCCCAAAGGGATGAGAAGTT-3′, 5′-CACTTGGTGGTTTGCTACGA-3′; IL-1β: 5′-AAATACCTGTGGCCTTGGGC-3′, 5′-CTTGGGATCCACACTCTCCAG-3′; MIP-2: 5′-AGACAGAAGTCATAGCCACTCTCAAG-3′, 5′-CCTCCTTTCCAGGTCAGTTAGC-3′; HPRT: 5′-CAGACTGAAGAGCTACTGTAATG-3′, 5′-CCAGTGTCAATTATATCTTCAAC-3′. Expression was normalized to hypoxanthine guanine phospho-ribosyl transferase (HPRT) levels and reported as the fold change in expression over the control group.

### 2.7. Western Blot Analysis

Lung tissue homogenate samples were separated on 6–8% SDS-polyacrylamide gels. Separated proteins were electrophoretically transferred onto nitrocellulose paper and blocked for 1 h at room temperature with Tris-buffered saline containing 3% bovine serum albumin. The membranes were probed with primary antibodies against cleaved caspase-3, phospho-Akt/Akt, or β-actin (1:1000 dilution) for 20 h followed by secondary antibodies (1:1000) for 30 min. Detection was performed using an enhanced chemiluminescence detection kit (Thermo Scientific).

### 2.8. Lung Histology, Histology Scoring, and TUNEL Staining

Lungs were fixed with 10% buffered formalin at room temperature for 48 h, dehydrated, and embedded in paraffin. Sections (4 μm thick) were stained with hematoxylin and eosin (H&E). Blinded analysis of lungs was performed using a light microscope. Lung pathological alterations were scored on a five-point scale according to the degree of neutrophil infiltration into airspace and peribronchiolar space of each image: (0) none (no neutrophil infiltration or a few neutrophils in several alveoli); (1) mild (a few neutrophils scattered in alveolar space in less than 10% of the field); (2) moderate (infiltration of neutrophils in alveolar space in 10–30% of the field); (3) severe (infiltration of neutrophils in alveoli in 30–60% of the field); (4) marked cellular infiltration (neutrophil infiltration in more than 60% of alveoli of the field) [43]. TUNEL staining was performed with a commercial kit (Roche, Bern, Switzerland) based on the manufacturer’s instructions. We observed apoptotic cells using a confocal microscope (LSM5 PASCAL; Carl Zeiss, Jena, Germany) equipped with a filter set with excitation at 488 and 543 nm. The quantitation of TUNEL-positive cells was performed by manually counting the number of TUNEL-positive cells per randomly selected 10 high-power fields (HPF) per section in a blinded fashion and averaged to give a single value per mouse.

### 2.9. Induction of Apoptosis

Human T lymphocyte Jurkat cells were obtained from the American Type Culture Collection (Rockville, MD, USA). Apoptosis was induced by ultraviolet irradiation at 254 nm for 10 min. The cells were then incubated for 2.5 h before use. They were 70% apoptotic by evaluation of nuclear morphology by light microscopy [36,37].

### 2.10. Ex Vivo Phagocytosis Assays

Jurkat T cells were fluorescently labeled with PKH67 (green) prior to apoptosis induction according to the manufacturer’s instructions (PKH67-Fluorescent Cell Linker Kits for General Cell Membrane Labelling; Sigma-Aldrich). In brief, 10^6^ Jurkat T cells/mL were washed in serum-free culture medium and resuspended in 2 mL PKH67-containing Diluent C (2 × 10^−6^ M) for 4 min at RT. Non-labeled alveolar macrophages (10^5^ cells/mL) from mice treated with saline, rGas6, BLM, or BLM+rGas6 were plated on coverslips in a 24-well plate. Then, PKH67-labeled apoptotic Jurkat cells were co-cultured with alveolar macrophages at a 10:1 ratio for 90 min at 37 °C in 500 μL DMEM. Coverslips were washed twice with phosphate-buffered saline (PBS) to remove the non-ingested AC. The slides were then fixed with 4% paraformaldehyde and permeabilized with 0.1% Triton X-100 (Sigma-Aldrich). The slides were mounted in Vectashield mounting medium with DAPI (Vector Laboratories, Inc., Youngstown, OH, USA). All slides were imaged using a confocal microscope (LSM5 PASCAL). For each condition, more than 200 alveolar macrophages were randomly observed and scored by two independent blinded observers. Each condition was tested in duplicate, and the reader was blinded to the sample identification during analysis. Efferocytosis of alveolar macrophages was determined using the phagocytic index.

### 2.11. In Vitro Exposure of BMDM, MLE-12, and A549 Cells to Stimulants

Primary BMDMs were isolated from C57BL/6 mice as previously described [44,45]. Briefly, BMDMs were differentiated using 20% L929 supernatant containing 10% fetal bovine serum (FBS) from murine bone marrow myeloid stem cells. After 7 days of culture, the differentiation of BMDM was confirmed by FACS analysis using anti-CD11b. BMDMs (10^6^ cells/mL) were pretreated with 400 ng/mL rGas6 before stimulating cytokine production with either LPS at 1 ng/mL or BLM at 5 μg/mL. Mouse lung type II epithelial cells (MLE-12) and A549 cells (adenocarcinomic human alveolar basal epithelial cell line) were obtained from ATCC (American Type Culture Collection, Manassas, VA, USA) and maintained in DMEM (GibcoTM, Thermo Fisher Scientific, Waltham, MA, USA) or RPMI 1640 (HyCloneTM, GE Healthcare, Logan, UT, USA) containing 10% FBS and 1% penicillin/streptomycin. For in vitro apoptosis experiments, cells were pretreated with 400 ng/mL rGas6 before stimulation with BLM at 50 μg/mL for 24 h.

### 2.12. In Vitro Apoptosis Assay

The annexin V-FITC/propidium iodide (PI) staining kit (BD Biosciences, San Jose, CA, USA) was used to detect apoptosis according to the manufacturer’s protocol. MLE-12 and A549 cells were harvested, resuspended in 150 mL binding buffer, and stained with 5 µL FITC-conjugated Annexin V and 5 µL PI in the dark for 15 min at room temperature. Cells positive for FITC-conjugated Annexin V were detected by flow cytometry (ACEA NovoCyte, San Diego, CA, USA). The data were analyzed using NovoExpress software 1.5. In addition, A549 cells were stained using the TUNEL kit (Roche, Basel, Switzerland), and apoptotic cells were observed using a confocal microscope.

### 2.13. Statistical Analysis

Data are expressed as mean ± SEM. Analysis of variance (ANOVA) was used to detect multiple comparisons, and Tukey’s post hoc test was applied where appropriate. The two-tailed Student’s *t* tests were used to detect significant differences between two sample means. A *p*-value of < 0.05 was considered statistically significant. All data were analyzed using Graph Prism 5 software (GraphPad Software Inc., San Diego, CA, USA).

## 3. Results

### 3.1. Administration of rGas6 Reduces Proinflammatory Cytokines and Enhances a Pro-Resolving Inflammatory Cytokine

**Figure 1:**
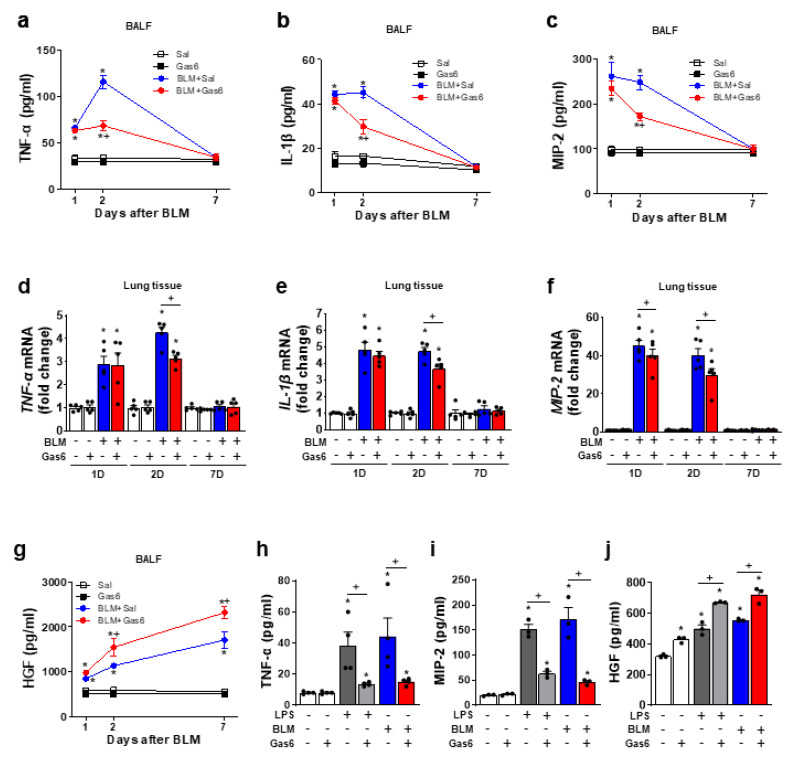
rGas6 reduces proinflammatory mediators but enhances HGF after bleomycin treatment in vivo and in vitro. (**a**–**g**) Bleomycin (BLM) was intratracheally instilled into mice. Either rGas6 (50 μg/kg) or saline was administered intraperitoneally 1 h before BLM treatment and every 24 h thereafter. Mice were sacrificed at 1, 2, and 7 days post−BLM instillation. (**a**–**c**,**g**) ELISA of TNF−α, IL−1 β, MIP−2, and HGF protein levels in the BAL fluid. (**d**–**f**) qPCR analysis of *TNF*−*α*, *IL*−*1β*, and *MIP*−*2* mRNA levels in lung tissue. (**h**–**j**) ELISA of TNF−α, MIP−2, and HGF protein levels in culture media of BMDMs pretreated with 400 ng/mL rGas6 before treatment with LPS (1 ng/mL) or BLM (5 μg/mL) for 24 h. The values represent the means ± SEM of results from five mice (**a**–**g**), four (**h**) or three independent in vitro experiments (**i**,**j**) in each group. * *p* < 0.05 compared with control. ^+^ *p* < 0.05 for BLM + Gas6 vs. BLM + Sal.

To evaluate the role of mouse rGas6 in BLM-induced lung inflammation, the protein levels of proinflammatory cytokines, including TNF-α, IL-1β, and MIP-2, were measured in BAL fluid using ELISA. We observed marked increases in these cytokines in BAL fluid at days 1 and 2 after BLM treatment; however, cytokine levels decreased to basal levels by 7 days post-treatment. Intraperitoneal administration of rGas6 (50 μg/kg/day) significantly suppressed BLM-induced TNF-α, IL-1β, and MIP-2 (approximately 58, 54, and 51% inhibition at 2 days post-BLM treatment, respectively) in BAL fluid (Figure 1a–c). Furthermore, the increased mRNA levels of TNF-α and IL-1β in lung tissue were also reversed by administration of rGas6 at 2 days post-BLM treatment (Figure 1d–f). We also detected an inhibitory effect of rGas6 on mRNA expression of MIP-2 at 1 and 2 days post-BLM treatment. The protein levels of the anti-inflammatory cytokine HGF in BAL fluid progressively increased up to 7 days after BLM (Figure 1g). The administration of rGas6 further enhanced the expression of HGF protein compared with the BLM + Sal group up to 7 days after BLM treatment.

The direct effects of rGas6 on pro- and anti-inflammatory cytokines were also evaluated in vitro. Treatment with 400 ng/mL rGas6 suppressed TNF-α and MIP-2 production in mouse BMDM cells stimulated with LPS (1 ng/mL) or BLM (5 μg/mL) (Figure 1h,i). rGas6 further enhanced HGF production in BMDMs stimulated with LPS or BLM. Similar to our in vivo findings (Figure 1j). These in vitro experiments suggest that rGas6 plays a resolving role in the inflammatory response in macrophages upon stimulation with BLM.

### 3.2. Administration of rGas6 Reduces Inflammatory Cell Recruitment and Total Protein Levels

**Figure 2:**
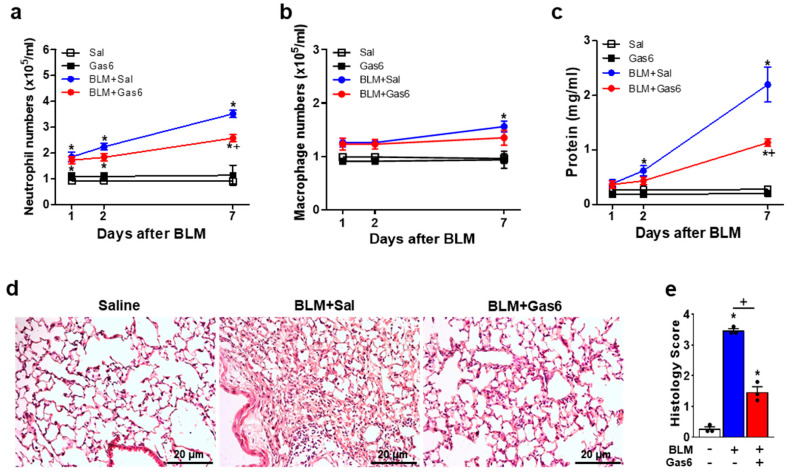
Administration of rGas6 attenuates bleomycin-induced inflammation. Bleomycin (BLM) was intratracheally instilled into mice. Either rGas6 (50 μg/kg) or saline was administered intraperitoneally 1 h before BLM treatment and every 24 h thereafter. Mice were sacrificed at 1, 3, and 7 days post−BLM instillation. (**a**,**b**) Neutrophil and alveolar macrophage numbers in BAL fluid. (**c**) Total protein levels in BAL fluid were analyzed by protein assay kit. (**d**) Hematoxylin–eosin stains of lung sections at 7 days after BLM (original magnification: ×200). Scale bars: all 20 μm. Representative images were obtained from three mice in each group. (**e**) Histology score of lung inflammation at 7 days after BLM treatment. The values represent the means ± SEM of results from five (**a**–**c**) or three mice (**d**,**e**). * *p* < 0.05 compared with saline control. ^+^ *p* < 0.05 for BLM + Gas6 vs. BLM + Sal.

Consistent with our previous studies [36,37], the recruitment of inflammatory cells, such as neutrophils and alveolar macrophages, into the lungs and total protein levels in BAL fluid progressively increased and peaked at 7 days post-BLM treatment. The administration of rGas6 significantly markedly reduced the number of neutrophils and the protein levels in BAL fluid at 7 days post-BLM treatment. Additionally, rGas6 weakly reduced alveolar macrophage numbers at 7 days post-BLM treatment (Figure 2a–c). Differential cell counts performed on BAL cytospin preparation at 7 days post-BLM treatment showed similar results of numbers of neutrophils and alveolar macrophages for each experimental group (data not shown). Since the maximal level of neutrophils in BAL fluid was observed on day 7, sections of lung tissue from these mice at 7 days post-BLM treatment were H&E stained. Histological findings revealed that rGas6 decreased the accumulation of inflammatory cells in alveolar spaces and lung parenchyma induced by BLM treatment. Furthermore, only mild alveolar structure destruction was present in lung tissues from mice administered with BLM and rGas6 compared with that from the BLM + Sal group (Figure 2d). Histological scores of lung inflammation were decreased significantly in the BLM + Gas6 group compared with the BLM + Sal group (Figure 2e).

### 3.3. rGas6 Inhibits Apoptosis in Lung Tissue and Alveolar Epithelial Cells In Vivo and In Vitro

**Figure 3:**
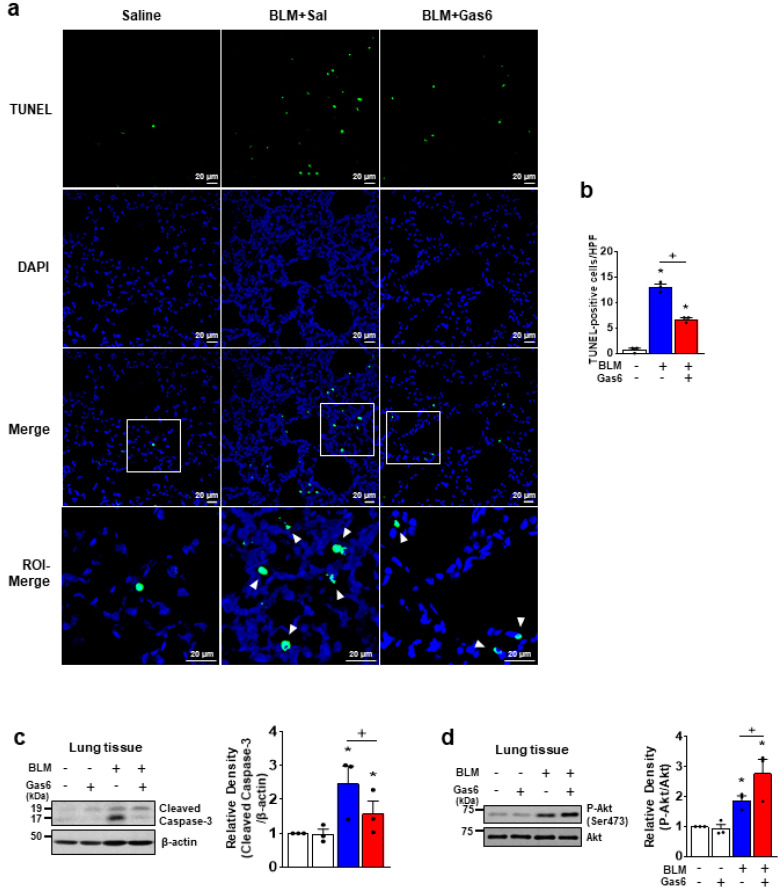
Administration of rGas6 inhibits bleomycin-induced apoptosis in lungs. Bleomycin (BLM) was intratracheally instilled into mice. Either rGas6 (50 μg/kg) or saline was administered intraperitoneally 1 h before BLM and every 24 h thereafter. Mice were sacrificed at 7 days post−BLM instillation. (**a**) Representative TUNEL−stained fixed tissue section (original magnification: ×200). Positive staining depicted in green. Nuclei were observed by staining. ROI−Merge panels show high magnification of ROIs, indicated in Merge panels as white squares. Scale bars = 20 µm. (**b**) Quantitation of the number of TUNEL−positive cells (number/HPF) in the different groups. (**c**,**d**) Immunoblot analysis of cleaved caspase−3, phosphorylated/total Akt, and β-actin in lung homogenates. Right: Densitometric analysis of each band and normalized to β-actin or Akt. The values represent the means ± SEM of results from three mice in each group. * *p* < 0.05 compared with saline control. ^+^ *p* < 0.05 for BLM + Gas6 vs. BLM + Sal.

We assessed DNA damage and apoptosis levels in lungs 7 days post-BLM treatment using the TUNEL assay and confocal microscopy. rGas6 administration reduced the number of TUNEL-positive lung cells per high-power field after BLM treatment (Figure 3a,b). In addition, Western blot analysis showed significantly decreased cleavage of caspase-3 in lung lysates from the BLM + Gas6 group compared with the BLM + Sal group (Figure 3c). The phosphorylation of anti-apoptotic kinase Akt in lung tissue was enhanced in the BLM + Gas6 group compared with the BLM +Sal group (Figure 3d).

**Figure 4:**
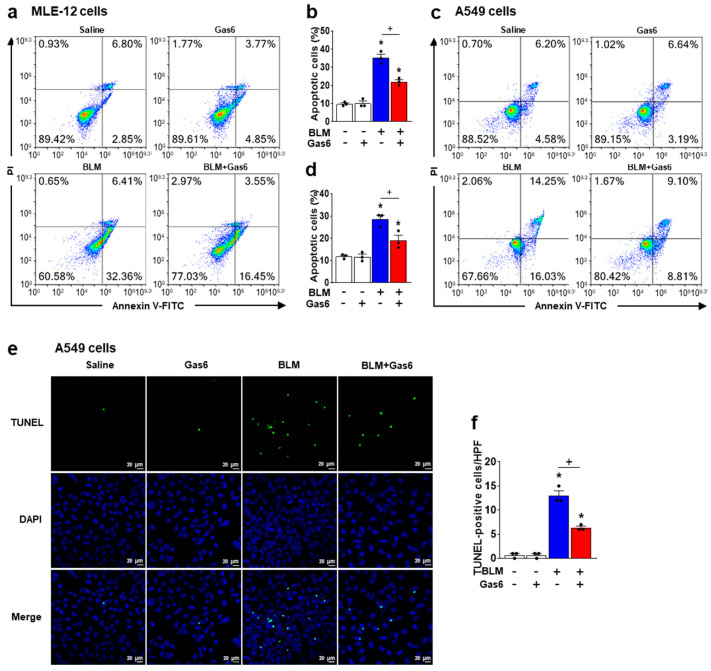
Treatment with rGas6 inhibits apoptosis in alveolar epithelial cells stimulated with bleomycin in vitro. (**a**,**b**) MLE−15 cells or (**c**–**f**) A549 cells were cultured and pretreated with 400 ng/mL rGas6 for 2 h before stimulation with BLM (50 μg/mL) for 24 h. (**a**–**d**) The cell viability was measured by flow cytometry after Annexin V−FICT/PI dual staining. (**b**,**d**) Apoptotic cells were quantified as the sum of the percentages of early and late stages of apoptosis. (**e**) Representative images of apoptosis in A549 cells by TUNEL assay (original magnification: ×200). Nuclei were observed by DAPI staining. Scale bar = 20 µm. (**f**) Quantitation of the number of TUNEL−positive cells (number/HPF) in the different groups. The values represent the means ± SEM of results from three independent experiments in each group. * *p* < 0.05 compared with control. ^+^ *p* < 0.05 for BLM + Gas6 vs. BLM.

To assess the direct inhibitory effect of rGas6 on the apoptosis of alveolar epithelial cells, we investigated whether rGas6 suppressed BLM-induced apoptosis of MLE-12 cells and A549 cells using Annexin V/PI staining and flow cytometry. Enhanced apoptotic cell numbers in MLE-12 (Figure 4a,b) and A549 cells (Figure 4c,d) after treatment with BLM were reduced by pretreatment with 400 ng/mL rGas6. Using the TUNEL assay in A549 cells, we also showed that the enhanced number of TUNEL-positive cells after treatment with BLM was markedly reduced by pretreatment with rGas6 (Figure 4e,f).

### 3.4. Administration of rGas6 Enhances Efferocytic Ability of Alveolar Macrophages during BLM-Induced Lung Inflammation

**Figure 5:**
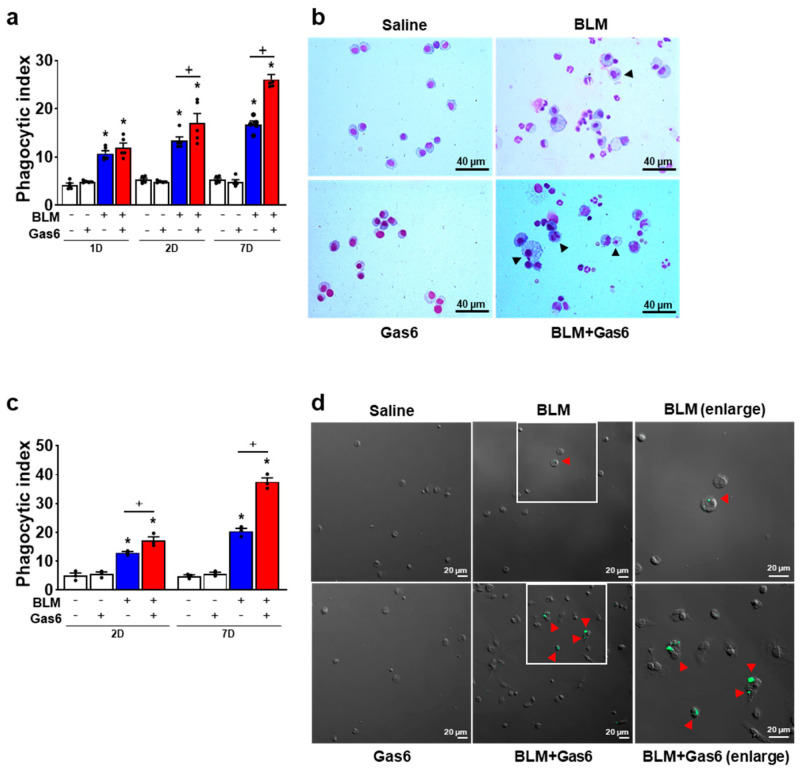
Administration of rGas6 reduces efferocytic ability of alveolar macrophages during bleomycin−induced lung inflammation. Bleomycin (BLM) was intratracheally instilled into mice. Either rGas6 (50 μg/kg) or saline was administered intraperitoneally 1 h before BLM treatment and every 24 h thereafter. Mice were sacrificed at 1 to 7 days post−BLM instillation. (**a**,**b**) Bronchoalveolar lavage (BAL) was performed, cytospins were stained, and alveolar macrophage ingestions of apoptotic cells were quantified by calculating a phagocytic index (PI). (**c**,**d**) Alveolar macrophages were cultured ex vivo with apoptotic Jurkat cells labeled with PKH67 (green) for 90 min, and phagocytosis was quantified by calculating a PI. Photomicrographs show (**b**) cytospin−stained BAL cells and (**d**) alveolar macrophages cultured ex vivo with apoptotic Jurkat T cells at 7 days post−BLM treatment. Arrowheads indicate alveolar macrophages with engulfed apoptotic cells or fragments. (**b**) Original magnification: ×400. Scale bars = 40 μm. (**d**) Green color represents apoptotic cells that are engulfed by alveolar macrophages. Original magnification: ×200. Scale bars = 20 μm. Values represent the means ± S.E.M. of results from five (**a**,**b**) or three mice (**c**,**d)** per group. * *p* < 0.05 compared with saline control. ^+^ *p* < 0.05 for BLM + Gas6 vs. BLM + Sal.

Endogenous apoptotic cells are generated and removed during an inflammatory reaction. After BLM treatment, it is likely that apoptotic neutrophils are the dominant cells being removed and that alveolar macrophages are globally activated [42]. Similar to the findings of our previous studies [36], the phagocytic indices in alveolar macrophages from mice treated with BLM alone were significantly enhanced compared with those from mice treated with control saline (Figure 5a,b). The administration of rGas6 further enhanced the efferocytic ability of alveolar macrophages at days 2 and 7 post-BLM treatment compared with the BLM + Sal group. In addition, the phagocytic activities of alveolar macrophages obtained at 2 and 7 days post-BLM treatment were assessed ex vivo. This approach has the advantage that it controls for the number and ratio of peritoneal macrophages to apoptotic cells [46]. Freshly obtained alveolar macrophages from saline, rGas6, or BLM- or BLM+rGas6-treated mice were co-cultured with apoptotic human Jurkat T cells labeled with PKH67 (green) to distinguish them from the endogenous apoptotic cells. The PI in the alveolar macrophages taken from BLM-treated mice was significantly enhanced ex vivo compared to saline controls at 2 and 7 days after BLM treatment (Figure 5c,d). The administration of rGas6 significantly further enhanced the ability of alveolar macrophages to phagocytose apoptotic cells ex vivo compared to those from BLM only-treated mice.

## 4. Discussion

BLM can cause ALI, which often results in pulmonary fibrosis due to a failure of the resolving acute inflammatory response. The pathology of the rodent BLM model of lung fibrosis includes acute alveolitis and interstitial inflammation, characterized by the recruitment of neutrophils and macrophages, with epithelial cell injury [6,47]. The role of Gas6 in the early stages of BLM-induced lung injury has not been characterized in detail. Together, previous studies from our laboratory and others support a role for Gas6 in inflammatory and immune responses mediated by Mer or Axl receptor tyrosine kinase [12,13,14,15,19,20,31,32]. rGas6 has been reported to inhibit TNF-α and IL-6 secretion by LPS-stimulated macrophages [13]. Gas6/Mer signaling can modulate macrophage cytokine secretion, triggering an anti-inflammatory pathway involving PI3K/Akt/GSK3β. In a mouse model of cecal ligation and puncture (CLP) sepsis, rGas6 treatment reduced lung serum levels of proinflammatory cytokines, such as IL-6 and IL-17, and mRNA levels of proinflammatory cytokines and chemokines, such as TNF-α, IL-1β, IL-6, IL-17, and MIP-2, at 20 h post-CLP [29]. Furthermore, rGas6 treatment attenuated the parenchyma of the lungs damaged by CLP. In a model of ex vivo ischemia/reperfusion-induced ALI, Gas6 directly modulated alveolar inflammation via up-regulating the phosphorylation of Axl and SOCS3 expression in lung tissue and the alveolar epithelium [31].

Here, we report that the administration of rGas6 attenuated BLM-induced lung inflammation and epithelial cell injury as well as enhanced the efferocytic ability of alveolar macrophages. Our data indicate that the administration of rGas6 before BLM treatment reduced the levels of proinflammatory cytokines, such as TNF-α, IL-1β, and MIP-2, in BAL fluid 2 days post-BLM treatment. rGas6 also markedly decreased the BLM-induced mRNA levels of these proinflammatory cytokines in lung tissue at 2 days post-BLM treatment. Additionally, production of the pro-resolving molecule HGF was further enhanced by rGas6 administration up to 7 days post-treatment compared with mice treated with BLM alone. Consistent with in vivo findings, we demonstrated that in vitro treatment with rGas6 inhibited TNF-α and MIP-2 production in mouse BMDMs stimulated with BLM or LPS, whereas HGF production was further enhanced in the same experimental condition. Of note, rGas6 enhanced HGF production by BMDMs compared with control. Previously, we also demonstrated rGas6-induced HGF mRNA expression and protein in the culture medium from RAW264.7 cells [48]. Our previous study also suggested that the Gas6-Mer receptor tyrosine kinase/RhoA/ERK and JNK signaling pathway were involved in HGF induction, consequently promoting epithelial cell growth and wound repair [26]. The administration of rGas6 resulted in a significant reduction in neutrophil recruitment into the lung and protein levels in BAL fluid at 7 days after BLM treatment. The enhanced resolution of inflammation was also confirmed by lung histology on day 7 after BLM treatment; lung histology showed a reduced infiltration of inflammatory cells in alveolus and lung interstitium. Collectively, in vivo and in vitro data from our studies suggest a suppressive role of Gas6 in the innate immune system, including macrophages and neutrophils.

Similar to the case of human idiopathic pulmonary fibrosis, apoptosis of pulmonary epithelial cells reflects the degree of lung injury and plays a key role in the initiation of the lung fibrotic process [49]. The inhibition of lung fibrosis by compounds with anti-apoptotic activity highlights the importance of apoptosis in the mechanism of tissue fibrosis [50,51]. Thus, suppressing epithelial apoptosis may serve as a treatment for ALI and may control the severe and irreversible progression of fibrosis. Intriguingly, compared with wild-type mice, protein S-transgenic mice have significantly decreased caspase-3 activity and an augmented expression of the apoptotic inhibitors BIRC5 and BIRC1b in their lungs [34]. In the present study, the increased expression of the activated (cleaved) form of caspase-3 in lung tissue and the number of TUNEL-positive lung cells in lung sections 7 days after BLM treatment were reduced by rGas6 administration. These changes were accompanied by increased phosphorylation of the anti-apoptotic kinase Akt. Furthermore, the anti-apoptotic effect of rGas6 was confirmed in alveolar epithelial cells in vitro. Here, we used MLE-12 (mouse) and A549 cells (human), because these cells have been used as in vitro models in previous (AT) II cell studies [52,53,54]. However, these two cell lines cannot completely reproduce the in vivo physiology of ATII cells. In future studies, primary cultured ATII cells may offer more direct evidence for the protective effect of rGas6. We speculate that rGas6 may prevent apoptosis of alveolar epithelial cells through the effect of Axl/Mer on down-regulating TNF-α and IL-1β expression [55,56] or up-regulating HGF production [57,58]. In addition to alveolar epithelial cells, exogenous administration of Gas6 suppressed LPS-induced TNF-α expression and apoptosis in H9C2 cardiomyocytes [59]. It is likely that Gas6 exerted an anti-apoptotic effect on vascular smooth muscle cells (VSMCs) [60]. These findings established the Gas6-Axl-PI3K-Akt pathway as an anti-apoptotic mechanism for cardiomyocytes and VSMCs.

Gas6 acts as a bridging molecule between apoptotic cells and phagocytes. Through the effective and efficient removal of apoptotic cells, inflammation can be reduced, and the harmful effects of secondary necrosis (leakage of cellular contents) can be abated [61,62]. Through this mechanism, Gas6 can dampen the inflammatory response to lung damage incurred by BLM administration. In addition, data from previous in vitro [63,64] and in vivo studies [36,37,65] demonstrated that increased efferocytosis of apoptotic cells by macrophages and epithelial cells enhances HGF and VEGF mRNA and protein levels. Thus, increased efferocytosis could promote the resolution of BLM-induced lung inflammation and fibrosis. Our data indicate that the administration of rGas6 further enhanced the efferocytic ability of alveolar macrophages during BLM-induced lung inflammation compared with the BLM + Sal group in vivo and ex vivo, which is associated with the down-regulation of neutrophil recruitment and total protein in BAL fluid. Of note, we demonstrated in a previous study that the anti-HGF antibody completely reversed the reduction of proinflammatory mediators, including TNF-α and MIP-2, neutrophil recruitment and total protein levels, as well as caspase-3 and -9 activities [36]. In parallel, data from the present study show that the pattern of time-dependent increases in HGF production and the efferocytic ability of alveolar macrophages is very similar following rGas6 treatment. Based on our previous and present findings, the mechanisms of the enhanced ability of alveolar macrophages to clear endogenous apoptotic cells appear to involve a mechanistic link of HGF induction and anti-inflammatory and anti-apoptotic effects of rGas6.

## 5. Conclusions

In summary, our data indicate that the administration of rGas6 reduced the levels of proinflammatory cytokines, such as TNF-α, IL-1β, and MIP-2, in BAL fluid and enhanced the levels of the anti-inflammatory cytokine HGF post-BLM treatment. rGas6 also suppressed neutrophil recruitment and protein levels in BAL fluid. The administration of rGas6 also resulted in a significant reduction in caspase-3 activity and apoptosis of lung cells. rGas6 treatment enhanced the phosphorylation of the anti-apoptotic kinase Akt in lungs post-BLM treatment. In addition, the efferocytic ability of alveolar macrophages was enhanced in mice administered both BLM and rGas6 compared with mice treated with BLM alone. Furthermore, in vitro, rGas6 promoted pro-resolving and anti-apoptotic effects in BMDMs and alveolar epithelial cells stimulated with BLM. Our findings suggest that rGas6 might be of therapeutic value for the resolution of lung injury after BLM treatment. These findings warrant further investigation using Gas6-deficient mice to characterize the regulatory role and signaling pathway of endogenous Gas6 in BLM-induced lung inflammation and apoptosis.

## Data Availability

All data are available within the manuscript and upon request to the corresponding author.
